# Primary bladder neck obstruction in men—new perspectives in physiopathology

**DOI:** 10.1038/s41391-023-00691-1

**Published:** 2023-07-08

**Authors:** Hannes Cash, Johann Jakob Wendler, Antonio Minore, Ioannis Kartalas Goumas, Luca Cindolo

**Affiliations:** 1 Clinic of Urology, Urooncology, Robotic and Focal Therapy of University Magdeburg, Magdeburg, Germany; 2 PROURO, Berlin, Germany; 3 Department of Urology, Università Campus Bio-Medico di Roma, Rome, Italy; 4 Department of Urology, Istituto Clinico Beato Matteo, Vigevano, Italy; 5 Department of Urology, Hesperia Hospital, CUrE Group, Modena, Italy

**Keywords:** Prostatitis, Medical research

Primary bladder neck obstruction (PBNO) is a dysfunction of the bladder neck (BN) in which the collum vesicae is narrow or fails to open adequately during voiding, resulting in bladder outlet obstruction [1]. The precise cause of PBNO isn’t fully understood, even if, historically, several etiologies have been hypothesized [2]. The clinical presentation of PBNO varies from voiding and storage symptoms, but also pelvic pain and discomfort, with or without ejaculation disorders [2–4]. Its incidence is underestimated but has been reported in 28–54% of young men (age 18–50) with lower urinary tract symptoms (LUTS) [2]. The common young patient with symptomatic PBNO has a low body mass index, limited comorbidity scores, lower PSA levels, and severe IPSS scores but with a small prostate volume [4–6]. A video urodynamic assessment with voiding cystourethrogram could show several degrees of urinary obstruction with adequate detrusor function without an adequate bladder neck opening [1, 2]. Endoscopically, the features considered suggestive of PBNO are lack of elasticity and hypercontraction of bladder neck (high bladder neck) in the absence of urethral strictures, posterior urethral valves, inflammatory lesions, or foreign bodies [3, 7]. Only very few studies specifically focus on the risk of developing long-term complications of PBNO (e.g., renal function, detrusor under activity). A better comprehension of the overall pathogenesis, the pathophysiological changes within the bladder neck over time and a broader awareness of PBNO as a differential diagnosis in younger men or men with a lifelong history of voiding disorder is needed. This also provides a basis to establish tailored therapies.

In this setting, excluding the embryological theories on bladder neck dysfunction, we focused on the critical evaluation of recently reported pathophysiological changes of the bladder neck leading to PBNO and proposed a new comprehensive hypothesis.

## Historical Findings and new theories

The structural changes at the BN causing PBNO have been described as “fibrous narrowing and hyperplasia” [7], “fibrous stroma and subepithelial scar, often with inflammatory change” [8], “abnormal morphologic arrangement of the bladder musculature (detrusor/trigonal)” [9]. Moreover, a structurally healthy but dysfunctional BN have also been described. Several theories included altered mechanisms of BN opening such as neurological dysfunction of the sympathetic nervous system with a detrusor-sphincter-dyssynergia [10], abnormal BN striated muscle components of the urethral sphincter [11], increase in the density of Neuropeptide Y immunoreactive nerves (supporting the hypothesis of the PBNO as a more functional illness linked to pelvic floor dyssynergia) [12].

To date, however, none of the hypothesized physiopathological mechanisms has reached a good reliability to impact clinical practice [13].

## Inflammatory pathway

Since 1959 [8], the role of inflammatory processes in PBNO has been theorized. We know that there is a relationship between prostatic inflammation and LUTS [14] and that physician-diagnosed prostatitis was associated with a 2.4-fold increase in the likelihood of a later diagnosed LUTS attributed to benign prostatic hyperplasia (BPH) [15]. More recently, possible links between prostatic inflammation and BN dysfunction have been published and gave renewed interest to this topic.

Analysing 30 periurethral prostatic tissues derived from whole prostates samples (prostate cancer specimen), Cantiello et al. showed that periurethral inflammatory infiltration was present in 70% of prostates. They described a significant correlation in terms of severity of IPSS to high collagenic deposition in patients with periurethral inflammation. The authors proposed that the periurethral fibrosis secondary to inflammation could cause LUTS through decreased urethral flexibility [16].

Fibbi in a large review on the role of the prostate as an immunocompetent organ, considered the presence of a bacterial and non-infectious chronic prostatitis, the initiating and inciting factors leading to tissue hyperproliferation. This change might be facilitated via the antigen-presenting capacity of prostatic stromal cells, which induce and sustain intraglandular immune responses. The authors supported the idea that the inflammation-induced damage of the prostatic tissue represents a chronic process of wound healing which activates hyperproliferative programs resulting in BPH nodules and collagenic dyssynergia deposition [17].

Robert et al. highlighted that prostatic inflammatory infiltrates have shown a wide spectrum of antigen-presenting cells involved in the maintenance of the sterility of the genital environment [18]. However, immune cells could also release cytokines and growth factors that recruit other cells that promote the growth of epithelial and stromal prostatic cells, with an unavoidable prostate volume enlargement and prostatic urethra compression [14].

PBNO-affected patients present symptoms which can be confounded with prostatitis, potentially leading to chronic pelvic pain [3] supporting the idea that PBNO can be also a consequence of previous prostatic inflammations. However, there is still a debate on the reversibility of subclinical prostatic inflammation. Wong examined the stability of the newly synthesized collagen in bacterial-induced prostatic inflammation and the reversibility of fibrosis and collagen content after resolution of infection and inflammation. Generating inflammation by injecting E.coli into prostates of mice the authors found the half-life of newly synthesized collagen to be significantly shorter in infected/inflamed prostates than in controls. Moreover, the authors found antibiotic treatment to reverse collagenic deposition, supporting that fibrosis linked to infectious disease is a reversible process [19]. Kim et al. demonstrated that profound epithelial mesenchymal transition is observed in lipopolysaccharide induced prostatitis and that the natural HIF-1α inhibitors ascorbate and curcumin were capable to attenuate prostate enlargement both in vivo and in vitro [20].

Prostatitis is known to be a frequently misdiagnosed condition, as young men who are affected by it, often don’t seek urological counseling. A sperm culture or Meares-Stamey tests are rarely prescribed, and a proper antibiotic or anti-infammatory treatment is rarely proposed. We hypotesized that the presence of all these conditions justify the persistence of a chronic inflammatory intraprostatic status. This lastly represents the promoter of progressive collagenic deposition and decrease of elastic system fibers that determine a sudden and long-term bladder outlet obstruction (due to a more rigid or less elastic BN and prostatic urethra that becomes more difficult to bend or compress) and LUTS progression [14, 21].

**Fig. 1:**
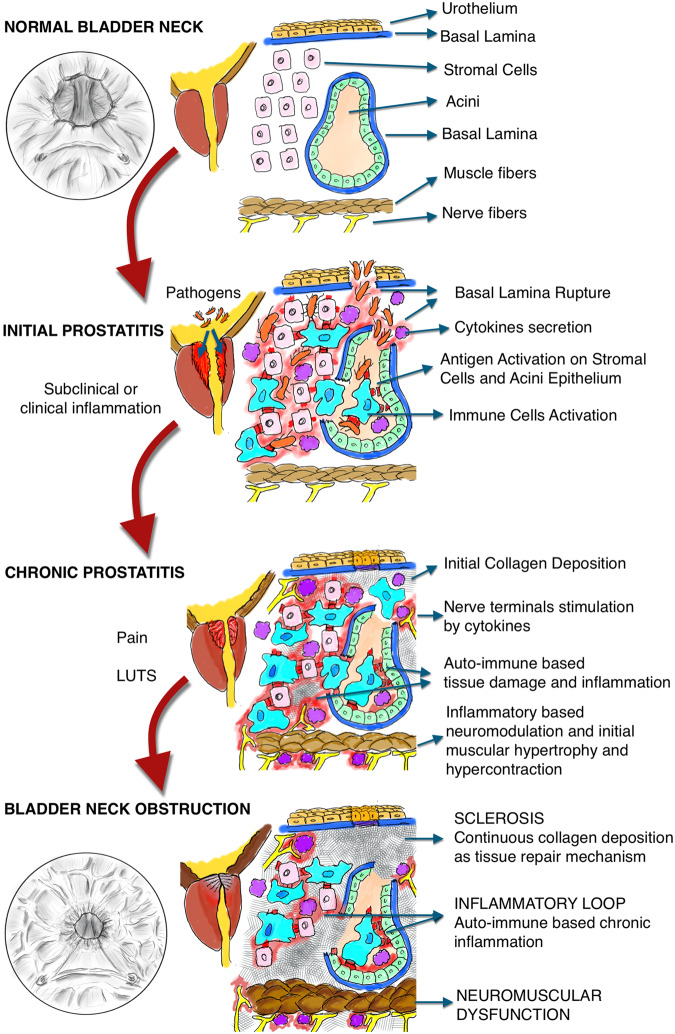
Pathophysiology of primary bladder neck obstruction. The hypotesized relationship between infection, inflammation and development of primary bladder neck obstruction is shown by the changes from the normal bladder neck, the initial inflammatory changes during prostatitis and the development of a chronic prostatitis. The continuous collagen deposition and underlying chronic inflammation lead to a bladder neck obstruction.

In other terms, we speculate on the possibility that the same inflammatory patterns that have been previously described for BPH development and BOO onset should be taken in consideration for the etiopathogenetic explanation of PBNO (Fig. 1).

The clinical value of these observations defines a comprehensive and precise diagnostic framework especially in young people referring pelvic pain, voiding and storage LUTS despite their small prostate volumes. The authors suggest that an accurate diagnosis could provide a timely treatment proposal that can stop symptomatic progression or, in some cases, reverse inflammation and collagenic deposition, limiting the risk of future obstruction.

## Non-inflammatory pathways

The data on the non-inflammatory possible causes of PBNO, are scarse and weak. Like Yalla [11], Billis [22] demonstrated a significantly amount of skeletal striated muscle fibers in transurethral resection of the prostate (TURP) resection fragments of the BN in PBNO patients than in patients with typical symptomatic BPH. In PBNO patients the fibers were thick, prominent, hypertrophied, and frequently in a parallel distribution; whereas BPH patients showed discrete, thin, and transversally or longitudinally cut fibers. They assumed that in PBNO there is a persistence of the cranial part of the skeletal muscle cells of the urethral sphincter, which may interfere in the complex micturition process, and that may explain why those patients did not respond to alpha-blockers, typically working on smooth muscle cells [22]. According to Yalla and Billis, Camarota and Zago hypothized, in a case series, a possible role of unbalanced biomechanics of the pelvis on the urethral and vesical sphincter activity by an unknown postural orthopedic condition with pelvic torsion that causes hypercontraction on the pelvic floor and interfering with the normal micturition [23, 24]. Camerota found out that PBNO patients have a high prevalence (76%) of myofascial or articular, mostly nociceptive pain across different regions as a relevant component [23]. They postulated that an interplay of peripheral inflammation, postural imbalance and chronic pain could induce nociceptor activation and sympathetic nervous system hyperactivation which in turn leads to a bladder sphincter dysfunction [23, 24] (Fig. 1). Hruz described in 60% of patients with diagnosis of category IIIb chronic pelvic pain syndrome a BN hypertrophy as the primary cause of their symptomatology [25].

Hinata described a correlation between increased prostate volume due to BPH and an increase of collagen fibers and degeneration of muscle bundles in the BN. By progression of prostatic hyperplasia, the BN muscles were progressively affected by fibrosis with fragmentation of the smooth muscle sphincter vesicae [26].

Bolton proved that BN shows a physiological variability around its circumference with a uniform response to noradrenaline of whole circumference, significantly stronger response to alpha-adrenergic agonists compared with cholinergic agonists in the posterior part, whereas the anterior part had no significant differential response to these [27, 28]. However, alpha-1-blocker just only have a variable success of 30–60% in PBNO.

## Discussion

A synopsis of the recent and sparse publications raises the suspicion of a multifactorial pathogenesis of PBNO with the result of infection/inflammation leading to degeneration of the BN and subsequent neuromuscular dysfunction. The differently described morphological characteristics and degrees of manifestation of PBNO and the different therapeutic response rates suggest a non-homogeneous and non-continuous remodeling process of the BN in PBNO. The different theories mentioned above do not necessarily contradict each other. Subacute or chronic prostatitis can be with no clinical signs of inflammation and no symptoms. LUTS can often be determined with variable characteristics and subjective perception. Likewise, in the case of pelvic pain syndrome, no clinical correlate can often be determined using conventional examination methods. However, this does not exclude occult inflammation with a corresponding pain reaction. According to clinical understanding, this can be accompanied by reactive changes in posture and movement, which in turn promote pain and stress reactions.

Currently, as of the 01/23/2023, no registered clinical study could be found with the term “Primary Bladder Outlet Obstruction” resp. “PBNO”. The search for “Bladder Outlet Obstruction” revealed 44 studies (ICTRP Search Portal Advanced Search (who.int); Search of: bladder outlet obstruction – List Results – ClinicalTrials.gov), with no study on PBNO or on the pathogenesis of PBNO being registered.

Further models of the inflammatory process and clinical studies are required to clarify the exact pathogenetic pathways of the PBNO development. The potential treatment options need to be tailored to the needs of the younger men presenting with symptomatic PBNO. With the importance of preservation of the ejaculatory function a wide range of new surgical minimally invasive therapies (MIST) are emerging [29–32]. The future challenge will be the identification of the perfect MIST for men suffering from PBNO.

In this perspective manuscript, we strongly propose that PBNO is an acquired disease and not a congenital anomaly. The degenerative remodeling process of the bladder neck based on an inflammatory change seems to be one of the potential drivers for symptomatic LUTS in young men.
